# Angiopoietin-1 promotes atherosclerosis by increasing the proportion of circulating Gr1^+^ monocytes

**DOI:** 10.1093/cvr/cvw223

**Published:** 2017-01-09

**Authors:** Takeshi Fujisawa, Keqing Wang, Xi-Lin Niu, Stuart Egginton, Shakil Ahmad, Peter Hewett, Christopher D. Kontos, Asif Ahmed

**Affiliations:** 1 Aston Medical Research Institute, Aston Medical School, Aston University, Birmingham B4 7ET, U.K;; 2 Gustav Born Centre for Vascular Biology and BHF Centre for Cardiovascular Sciences, University of Edinburgh, Edinburgh EH16 4TJ, UK;; 3 Department of Medicine, Division of Cardiology, Duke University Medical Centre, Durham, NC 27710, USA;; 4 Multidisciplinary Cardiovascular Research Centre, School of Biological Sciences, University of Leeds, Leeds, UK;; 5 Institute of Cardiovascular Sciences, College of Medical and Dental Sciences, University of Birmingham, Birmingham, UK

**Keywords:** Angiopoietin-1, Atherosclerosis, Monocytes

## Abstract

**Aims:**

Atherosclerosis is a chronic inflammatory disease occurring within the artery wall. A crucial step in atherogenesis is the infiltration and retention of monocytes into the subendothelial space of large arteries induced by chemokines and growth factors. Angiopoietin-1 (Ang-1) regulates angiogenesis and reduces vascular permeability and has also been reported to promote monocyte migration *in vitro*. We investigated the role of Ang-1 in atherosclerosis-prone apolipoprotein-E (Apo-E) knockout mouse.

**Methods and results:**

Apo-E knockout (Apo-E^-/-^) mice fed a western or normal chow diet received a single iv injection of adenovirus encoding Ang-1 or control vector. Adenovirus-mediated systemic expression of Ang-1 induced a significant increase in early atherosclerotic lesion size and monocyte/macrophage accumulation compared with control animals receiving empty vector. Ang-1 significantly increased plasma MCP-1 and VEGF levels as measured by ELISA. FACS analysis showed that Ang-1 selectively increased inflammatory Gr1^+ ^monocytes in the circulation, while the cell-surface expression of CD11b, which mediates monocyte emigration, was significantly reduced.

**Conclusions:**

Ang-1 specifically increases circulating Gr1^+ ^inflammatory monocytes and increases monocyte/macrophage retention in atherosclerotic plaques, thereby contributing to development of atherosclerosis.

## 1. Introduction

Atherosclerosis is a chronic inflammatory disease of the artery wall.^1–3^ Monocyte-derived macrophages participate in a maladaptive, non-resolving inflammatory response that expands the subendothelial layer due to the accumulation of cells, lipid and matrix.^4^ Signals such as monocyte chemoattractant protein-1 (MCP-1) increase monocyte recruitment into atherogenic foci,^5^^,^^6^ in particular, Ly6C^high^(Gr1^+^) inflammatory monocytes that gives rise to macrophages in atheromata.^7^

Angiopoietin-1 (Ang-1) and its cognate receptor Tie2 are well-established regulators of vascular development and angiogenesis.^8–10^ Ang-1 plays a crucial role in endothelial cell survival, vessel wall remodelling and mural cell recruitment.^11^ Overexpression of Ang-1 dramatically blocks increases in vascular permeability induced by VEGF,^12^^,^^13^ suggesting that it has protective properties in the microvasculature.^14^ However, long-term Ang-1 expression using adeno-associated virus failed to protect against the development of rat cardiac allograft arteriosclerosis.^15^ Jeansson *et al.*^8^ recently reported that Ang-1 is not necessary for normal steady-state physiological processes in the adult, being expendable in the blood vasculature from E13.5 onwards,^10^ but when combined with injury, Ang-1 deficiency results in accelerated angiogenesis and fibrosis.^10^ Long *et al.*^14^ have reported that Ang-1 therapy accompanied pro-fibrotic and inflammatory effects in folic acid-induced tubular necrosis and in a murine model of acute renal injury.^16^ More importantly, Ang-1 is expressed at a higher level in atherosclerotic lesions obtained from endarterectomy of the carotid artery compared to healthy controls.^17^ Furthermore, Ang-1 stimulates TNF-α, a key cytokine that modulates the inflammatory process of atherosclerosis.^18^ expression in peripheral blood mononuclear cells,^19^ Indeed, we and others have demonstrated that Ang-1 stimulates monocyte^20^ and neutrophil^21^ migration, both of which are the critical players in atherosclerosis,^22^^,^^23^ implicating Ang-1 as a potential player in monocyte recruitment and retention mechanisms especially in a high-lipid environment.

Based on these observations, we hypothesized that Ang-1 plays a role in the progression of atherosclerosis in a hypercholesterolaemic environment through its effects on inflammatory monocytes. In this study, we demonstrate that high circulating Ang-1 levels promote a pro-atherogenic phenotype by specifically increasing Gr1^+ ^inflammatory monocytes and elevating the circulating levels of pro-remodelling cytokines, VEGF and MCP-1 in ApoE^-/-^ mice. Furthermore, Ang-1-induced monocyte/macrophage retention in atherosclerotic plaques was accompanied by decreased cell-surface expression of CD11b on circulating monocytes.

## 2. Methods

### 2.1 Adenoviruses

Recombinant adenovirus encoding human Ang-1* (AdAng-1) was provided by Regeneron Pharmaceuticals (Tarrytown, New York, USA), propagated in Human embryonic kidney cells 293 (HEK293), purified on CsCl gradients, titered, and stored at −80 °C in 4% sucrose buffer. Control, empty adenovirus (AdEV) was generated as described previously.^22^

### 2.2 Adenovirus-mediated expression of Ang-1 in ApoE^-/-^ mice

All procedures conformed to the recommendations of the Guide for the Care and Use of Laboratory Animals published by the US National Institutes of Health (NIH Publication, 8th Edition, 2011) and also in accordance with Directive 2010/63/EU of the European Parliament and with the UK Home Office Animal (Scientific Procedures) Act 1986. All procedures passed local ethical review. Eight- to nine-week-old male **Apo**E^**-/-**^ mice on a C57BL/6J background (B6.129P2-*apoE^tm^*^1^*^Unc^*, SN: 002052; Jackson Labs, Maine, USA) were maintained with a 12-hour light/dark cycle and had free access to food and water. To investigate the effects of Ang-1 on atherosclerosis under high-fat environment, the mice were fed a western-style diet (TD 88137; Harlan Teklad, South Easton, MA, USA) for 1 week and then divided into two groups (*n* = 12 per group) and injected via tail vein with 5 × 10^9^ pfu of AdAng-1 or control empty virus (AdEV) diluted into 100 μL PBS. These mice were maintained on a western diet for another 4 weeks. In another sets of experiments, mice fed a normal chow diet (*n* = 5 in each group in each experiment). These mice were injected with 5 × 10^9^ pfu of AdAng-1 or control empty virus (AdEV) diluted into 100 μL PBS through tail vain. The mice were euthanized 4 weeks after virus delivery. Briefly, cardiac puncture was performed with 2.3% isoflurane inhalation, and blood was collected in EDTA tube. Mice were injected with Pentabarbitone (Euthatal, 270 mg/kg, ip injection) to euthanize and then perfuse-fixed with 1% paraformaldehyde. Heart and proximal aortae were harvested. Serial 5 µm frozen sections of the aortae were prepared as described previously.^24^ For the evaluation of atherosclerotic lesions, nine sections were taken at 40 µm intervals, stained with oil red O, counterstained with Mayer’s hematoxylin, and the lesions were quantified using NIH Image software (v. 1.62). The mean area in nine sections was determined for each animal. Up to three sections from each animal were immunostained for monocytes/macrophages using a monoclonal rat anti-mouse monocyte/macrophage antibody (MOMA-2, BD PharMingen*)* and quantification of MOMA-2-positive area was performed. The mean MOMA-2-positive area was determined for each animal. The aortic arches were available from some animals and Sudan IV *en face* staining was performed for analysis of lipid accumulation, as described elsewhere.^25^ Blood was taken at 3 and 10 days after virus injection by tail bleed and at 28 days by cardiac puncture.

### 2.3 Aortic ring culture

Male ApoE^-/-^ mice (8–10 weeks old) were euthanized and bled out. Thoracic aortas were removed into a Petri dish filled with cold sterile PBS and aortas were mechanically cleaned of surrounding fat tissue. Using a surgical blade, aortas were evenly cut into 1 mm rings, which were transferred to fresh DMEM medium supplemented with 1% fetal calf serum (FCS), penicillin and streptomycin. Aortic rings were stimulated with Ang-1 (400 ng/mL; R&D Systems, Abingdon, UK) for 24 hours at 37 °C. Some of rings were pre-incubated with Tie2 blocking peptide^26^ (NLLMAAS, 100 µM; Peptide Protein Research Ltd, Hampshire, UK) for 30 min prior to stimulation with Ang-1. Each condition was performed in duplicates. Commercial ELISA as described below measured the levels of MCP-1 and VEGF in the conditioned medium.

### 2.4 ELISA assay

Whole blood was collected into EDTA-containing tubes. ELISA was used to measure plasma levels of Ang-1, MCP-1 and VEGF (R&D Systems).

### 2.5 Immunohistochemistry

Immunohistochemistry was performed on serial frozen sections. Primary rat anti-mouse antibodies to monocyte/macrophage (MOMA-2), and CD31 were from BD Pharmingen. Isotype-matched non-binding immunoglobulin was used as a negative control. Binding of secondary antibodies (Vector Laboratories, Peterborough, UK) was detected with Vectastain ABC reagent (Vector Laboratories, Peterborough, UK) and DAB substrate kits (Dako, Cheadle, UK). Cells were counterstained with Mayer’s hematoxylin.

### 2.6 Flow cytometry

Whole-blood samples (15 μL) were washed with cold PBS before incubating for 20 min on ice with directly conjugated antibodies: anti-Ly6C-APC (eBioscience, Cheshire, UK), anti-CD11b (eBioscience, Cheshire, UK) and anti-Ly6G (Gr1)-FITC (eBioscience, Cheshire, UK). Monocytes were gated according to CD11b expression and side scatter and then further separated by Gr1 and Ly6C staining. Two monocyte subsets were identified as SSC^low^CD11b^+ ^Gr1 ^+^ Ly6C^high^ and SSC^low^CD11b^+ ^Gr1^-^Ly6C^low^ monocytes, whereas neutrophils were identified as SSC^high^CD11b^+ ^Gr1^high^Ly6C^inte^ (*Figure 3A and B*) using FACSCalibur. Combination of anti-CD11b, anti-Gr1 and anti-Tie2-PE (eBioscience, Cheshire, UK) were used to stain cells to analyse Tie2-positive monocyte population.

### 2.7 Measurement of plasma Ang-1

Blood was collected from mice at days 0, 3, 7, 14, 21, and 28 after injection of AdEV (*n* = 3) or AdAng-1 (*n* = 3), mixed with EDTA. Plasma levels of Ang-2 were determined a using specific ELISA following the manufacturer’s instructions (R&D Systems, MN, USA).

### 2.8 Confocal immunofluorescence staining

Tissue sections were stained with rat monoclonal anti-Ly6C (IgG2c; eBioscience, Paisley, UK) followed by Alexa Fluor 568-conjugated anti-rat IgG (Invitrogen, Paisley, UK). Then counter stained with anti-CD11b-FITC (eBioscience, Cheshire, UK) followed by goat anti-FITC-conjugated with Alexa Fluor488 (Invitrogen, Paisley, UK). Sections were washed with PBS, coverslip mounted and imaged on a Leica SP5C inverted confocal laser scanning microscope.

### 2.9 Statistical analysis

Results are expressed as the mean ± SEM. Comparisons between two groups were performed using unpaired Student’s *t*-tests, and comparisons among multiple groups were performed using One-way Analysis of variance (ANOVA). Statistical analyses were performed using Prism 7.0 (GraphPad Software, Inc., La Jolla, CA, USA). *P* values^ ^<0.05 were considered statistically significant.

## 3. Results

### 3.1 Ang-1 enhances atherosclerotic plaque formation in ApoE^-/-^ mice fed a western diet

**Figure 1:**
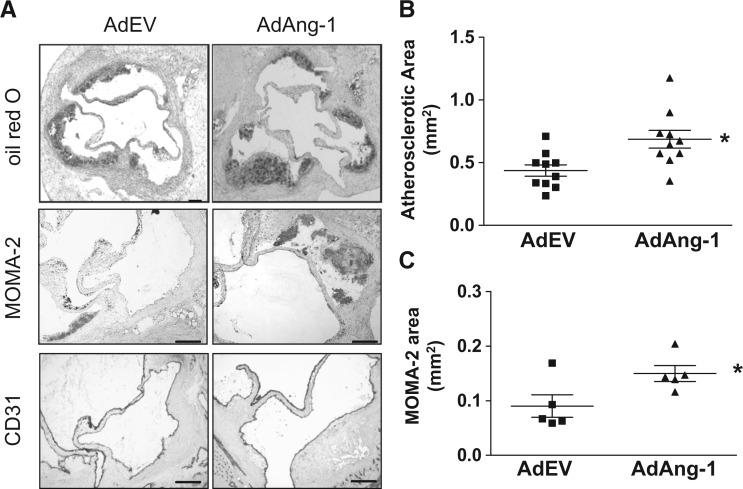
Ang-1 enhances atherosclerotic plaque formation in ApoE^-/-^ mice fed a Western diet. ApoE^-/-^ mice fed a Western diet for 4 weeks were infected systemically with adenoviruses encoding Ang1 (AdAng-1) or a control virus (AdEV). The mice were euthanized 4 weeks after adenovirus treatment and effects on early to intermediate atherosclerotic lesions were investigated. (*A*) Representative sections stained with oil red O (top panel) and immunohistochemical staining of monocytes/macrophages and endothelium with the MOMA-2 monoclonal antibody (middle panel) and CD31 antibody (bottom panel) respectively in AdEV or AdAng1-treated ApoE^-/-^ mice. Original magnification, ×40 (Oil red), ×100 (MOMA-2). Bars, 100 µm. (*B*) Atherosclerotic lesion area was quantified by oil red O staining of lesions from serial aortic sections using ImageJ image analysis software. Nine serial sections at 40 µm intervals were used from each animal for analysis. Results show the mean from nine cross-sectional lesion size (mm^2^) for each animal and the line indicates median value per treatment of mice. *P < 0.01. (*C*) Similarly, five mice were randomly picked from each treatment group, and tissue sections from each animal were analysed for MOMA-2-positive lesion area. *P<0.05.

To investigate the effect of Ang-1 on atherosclerosis development, ApoE^**-/-**^ mice fed a western diet for 1 week were given AdAng-1 or control AdEV injection and maintained on a western diet for a further 4 weeks. It has been demonstrated that iv delivery of adenovirus leads to a widespread distribution of vector with the highest level of expression in the liver.^27^ The level of Ang-1 in plasma peaked 3 days after injection and remained elevated for >10 days (Supplementary material online, *Figure S1*). No significant differences in body weight or circulating lipid profiles were detected between these groups (Supplementary material online, *Table S1*). Four weeks after systemic adenovirus administration, atherosclerotic lesions were analysed in aortas sections using oil red O staining (*Figure 1A*). Systemic overexpression of Ang-1 significantly increased the mean atherosclerotic lesion size compared with AdEV-infected ApoE^**-/-**^ mice (0.687 ± 0.071 mm^2^ vs. 0.437 ± 0.045 mm^2^; *P *<* *0.01; *Figure 1B*). Uninfected ApoE^**-/-**^ mice had lesions similar in size to the AdEV-infected group (0.547 ± 0.05 mm^2^, data not shown). In addition, with macrophages being a primary cell type contributing to development of the atherosclerotic lesion, we examined the effect Ang-1 on monocyte/macrophage accumulation in atherosclerotic lesion using monocyte/macrophage marker MOMA-2. As expected, Ang-1 induced a significantly increased monocyte/macrophage accumulation in lipid lesions compared with control animals receiving empty vector (0.15 ± 0.015 mm^2^ vs. 0.09 ± 0.021 mm^2^; *P *<* *0.05; *Figure 1C*). Endothelium staining using CD31 antibody showed no significant vascularization in aortas (*Figure 1A*)

### 3.2 Ang-1 promotes atherosclerotic plaque formation in ApoE^-/-^ mice fed a normal chow diet

**Figure 2:**
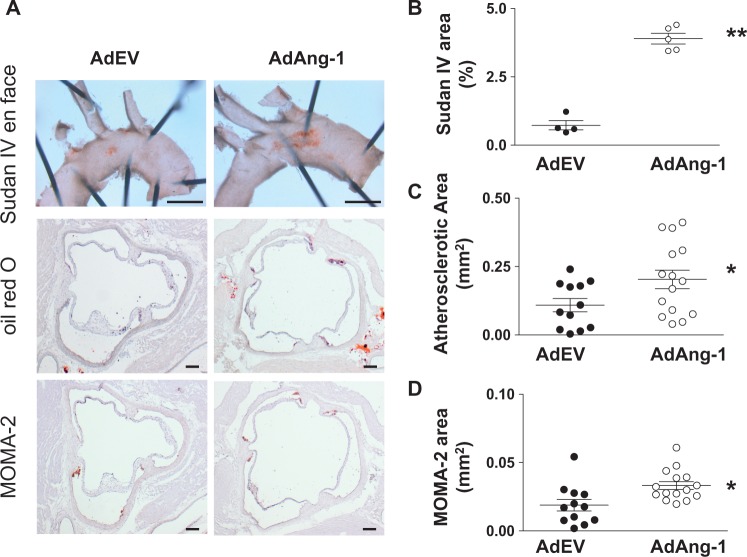
Ang-1 promotes early atherosclerotic plaque formation in ApoE^-/-^ mice fed a normal chow diet. ApoE^-/-^ mice fed a normal chow diet were infected systemically with adenoviruses encoding Ang-1 (AdAng-1), or control virus (AdEV). The mice were euthanized after 4 weeks of adenovirus treatment and effects on early to intermediate atherosclerotic lesions was investigated. (*A*) Representative picture of Sudan IV *en face* (top panel) (n = 4 per group). (*B*) Sudan IV *en face* staining was quantified from the beginning of aortic arch to the left common carotid artery using image analysis software and expressed as the percentage lesion area in each vessel. **P < 0.001. (*C*) Oil red O-positive atherosclerotic areas were calculated by using ImageJ image analysis software (*n* = 12–15 per group). Atherosclerotic plaques were quantified as described in the legend to *Figure 1*. *P<0.05. (*D*) Similarly, three sections from each animal were analysed for MOMA-2-positive lesion size (mm^2^) (*n* = 12–15 per group). *P<0.05. Original magnification ×2.5 (Sudan IV *en face*); bars, 1 mm (Sudan IV).

In order to further confirm the effects of Ang1 in atherosclerosis development without the confounding effects of western diet on inflammation in apoE^-/-^ mice, AdAng-1 or control AdEV were injected in apoE^-/-^ mice fed a normal chow diet and *en face* analyses of Sudan IV-stained areas in thoracic aortas were quantified 4 weeks after treatment. Ang-1 significantly increased plaque area compared with AdEV-treated ApoE^-/-^ mice (3.90 ± 0.195 mm^2^ vs. 0.73 ± 0.169 mm^2^; *P *<* *0.01; *Figure B*). Oil red O-positive atherosclerotic area and MOMA-2-positive area were also analysed as before (*Figure D*). As expected, mice fed a normal chow diet developed much smaller plaques compared with mice on a western diet. However, the plaques were again significantly larger in the AdAng-1-treated group compared to the AdEV-treated group (0.20 ± 0.034 mm^2^ vs. 0.11 ± 0.024 mm^2^; *P *<* *0.05; *Figure 2C*). Similarly, the accumulation of MOMA-2-positive cells, which co-localized with oil red O staining in the aortic root (*Figure 1A*), was also increased in the AdAng-1-treated group (0.03 ± 0.003 mm^2^ vs. 0.02 ± 0.004 mm^2^; *P *<* *0.01; *Figure 2D*)

### 3.3 Ang-1 increases proportion of circulating Gr1^+^/Ly6C^high^ monocytes

**Figure 3:**
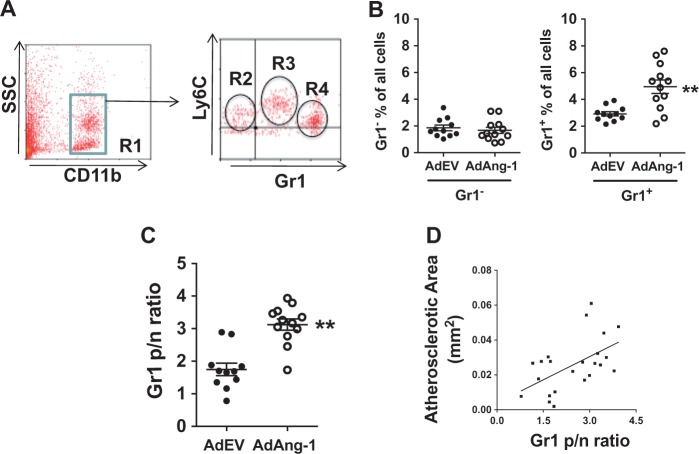
Ang-1 increases circulating numbers of Gr1^+ ^monocytes. Whole-blood samples were stained with CD11b, Gr1 and Ly6C antibodies and analysed by flow cytometry. (*A*) CD11b-positive cells were gated (R1) according to CD11b expression and side scatter. R1 cells were further divided into SSC^low^CD11b^+^Gr1^-^Ly6C^low^ monocytes (R2) and SSC^low^CD11b^+^Gr1^+^Ly6C^high^ monocytes (R3) and SSC^high^CD11b^+^ Gr1^high^Ly6C^inte^ neutrophils (R4). (*B*) 10 days after virus administration, the proportions of Gr1^-^ and Gr1^+^ moncoytes in each treatment group was calculated as the percentage of total white blood cells. (*C*) To correct for variability among individual experiments, the Gr1^+^/Gr1^-^ ratio was calculated for each treatment group. (*D*) The correlation between MOMA-2-positive areas and Gr1^+^ and Gr1^-^ monocytes on day 10 after AdAng-1 administration was analysed. Pooled data are presented from three separate independent experiments (*n* = 4–5 per experiment). **P<0.01.

**Figure 4:**
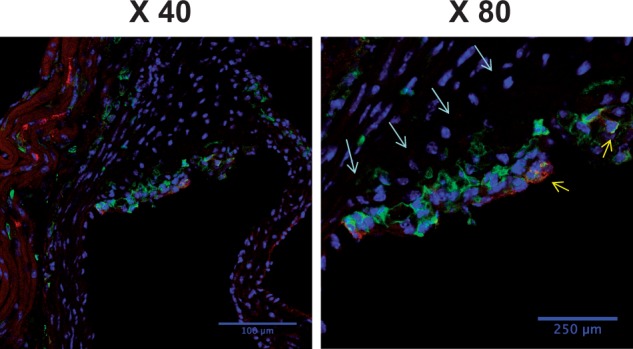
Ang-1 induces Ly6C ^+^ CD11b^+ ^monocytes accumulation in *ApoE^-/-^* mice fed a normal chow diet. Representative immunofluorescence staining of Ly6C and CD11b monoclonal antibodies in AdAng1-treated ApoE^-/-^ mice. Red: Ly6C, green: CD11b, blue: 4’,6-diamidino-2-phenylindole (DAPI). Original magnification, ×40 (left), ×80 (right). White arrow is the border line between arterial wall and plaque judging by out layer of muscle filament. Yellow arrow is double staining mononuclear cell.

It is widely accepted that bone marrow-derived circulating monocytes play an important role in atherosclerosis^28^ and that different subsets of monocytes commit for specific functions while still in the circulation.^29^ Particularly, Ly6C^high^(Gr1^+^) inflammatory monocytes give rise to macrophages in atheromata.^7^ To investigate the role of Ang-1 on the dynamics of monocyte turnover/recruitment, peripheral monocytes in ApoE^-/-^ mice fed a normal chow diet were evaluated at different time points after AdAng-1 treatment (3, 10 and 28 days). Monocytes were gated according CD11b^+ ^and SSC (*Figure 3A* R1) and two monocyte subsets were identified as SSC^low^CD11b ^+^ Gr1 ^+^ Ly6C^high^ (*Figure 3A* R3) and SSC^low^CD11b ^+^ Gr1^-^Ly6C^low^ monocytes (*Figure 3A* R2), which correspond to inflammatory and residential subsets, respectively.^30^ A third CD11b^+ ^cells population with high SSC, low Ly6C are neutrophils (*Figure 3A* R4).^7^^,^^30^ The proportion of circulating Gr1^+ ^monocytes in AdAng-1-treated mice was significantly increased at day 10 compared with AdEV-treated mice (4.95 ± 0.503 *vs*. 2.93 ± 0.163; *P *<* *0.01), whereas Gr1^-^ monocytes remained unchanged (*Figure 3B*). Interestingly, neutrophil proportion was also increased (9.53 ± 3.92 vs. 13.74 ± 4.04; *P* < 0.05). Significant increase in the ratio of Gr1^+^/Gr1^-^ monocytes (*Figure 3C*) was positively correlated with plaque size (*Figure 3D*), indicating the importance of monocyte subsets balance in disease progression. At day 3 and day 28, the proportions of Gr1^-^ and Gr1^+ ^monocytes showed no differences between AdEV and AdAng-1 treated groups (data not shown). Furthermore, immunostaining of aorta obtained from ApoE^-/-^ mice received AdAng-1 treatment revealed that Ly6C-positive cells localized in the plaques (*Figure 4*) in line with the current understanding that this group of cells is likely to account for the observed accumulation of monocytes/macrophages in the plaques.

It is believed that inflammatory monocytes derived from bone marrow play a crucial role in atherogenesis. Recently, spleen has been identified as a site for storage and rapid deployment of monocytes, which contribute to atherosclerosis development.^31^ Interestingly, preliminary study showed that proportion of bone marrow Ly6C^high^ monocytes was reduced following AdAng-1 treatment, whereas splenic Ly6C^high^ monocytes remained unchanged (data not shown), suggesting that Ly6C^high^ inflammatory monocytes are likely mobilized from bone marrow in response to Ang-1.

### 3.4 Ang-1 increases plasma levels of VEGF and MCP-1 in ApoE^-/-^ mice fed a normal chow diet

**Figure 5:**
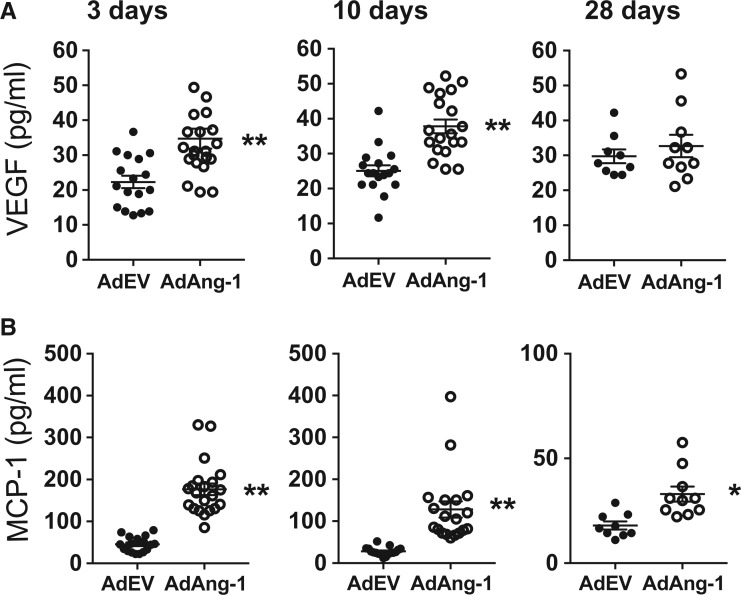
Ang-1 increases plasma levels of VEGF and MCP-1 *ApoE^-/^*- mice fed a normal chow diet. Whole blood was collected at 3 and 10 and 28 days after AdEV or AdAng-1 administration and plasma was isolated. ELISA was used to measure the level of VEGF (*A*) and MCP-1 (*B*). Pooled data are presented from four independent experiments (*n* = 17–19 per group). **P <* *0.01.

Chemokines such as MCP-1 is crucial for monocyte mobilization from bone marrow.^32^ To test whether overexpression of Ang-1 alters circulating cytokine/chemokine profiles, we analysed the plasma concentrations of VEGF and MCP-1 by ELISA. Ang-1 significantly increased both plasma VEGF (*Figure 5A*) and MCP-1 (*Figure 5B*) levels on day 3 and 10 after administration of viruses. Notably, the increased level of MCP-1 persisted even on day 28 when plasma Ang-1 concentrations had subsided (Supplementary material online, *Figure S1*). Furthermore, the plasma level of VEGF significantly correlated with expression of MCP-1 on day 10 in AdAng-1-treated but not in AdEV-treated animals (Supplementary material online, *Figure S2*).

### 3.5 Ang-1 induces release of VEGF and MCP-1 from aortic rings

**Figure 6:**
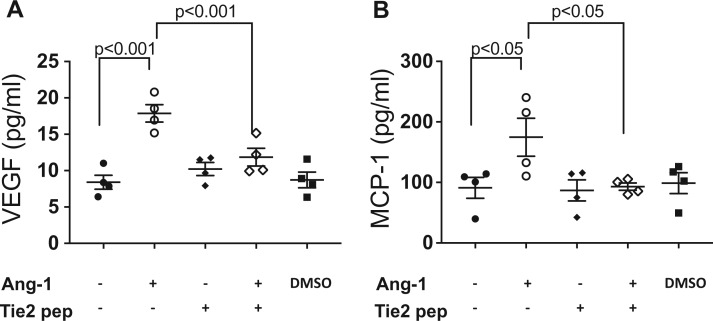
Ang-1 induces the VEGF and MCP-1 release in conditioned medium from aortic ring. Thoratic artery from apoE^-/-^ mouse was cut into 1 mm segments and aortic rings were stimulated with Ang-1 (400 ng/mL) with or without pre-incubation with Tie2 blocking peptide (NLLMAAS, 100 µM) for 30 min. Medium containing 0.01% DMSO was served as vehicle control. The levels of VEGF (*A*) and MCP-1 (*B*) in conditioned medium were measured by ELISA assay. Pooled data are presented from four independent experiments performed in duplicates.

Next, we performed aortic ring cultures to investigate the origin of VEGF and MCP-1 upon stimulation with Ang-1. Ang-1 significantly increased release of both VEGF and MCP-1 from aortic rings, and these effects were blocked by pre-incubation with the Tie2 peptide (*Figure 6*), suggesting that Ang-1 induces vascular cell expression of VEGF and MCP-1, thereby creating a pro-remodelling environment in large vessels in which atherosclerotic plaques form. In addition, expression of VEGF and MCP-1 was significantly correlated with one another in these aortic rings (Supplementary material online, *Figure S3*).

### 3.6 Ang-1 reduces cell-surface expression of CD11b on Gr1^+ ^monocytes

**Figure 7:**
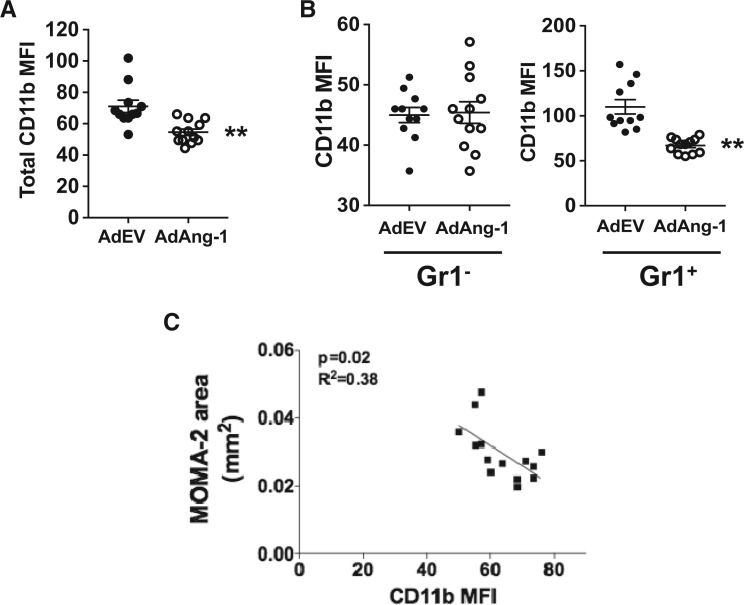
Ang-1 reduces cell-surface CD11b expression on Gr1^+ ^monocytes. Whole-blood samples were stained with anti-CD11b and anti-Gr1 antibodies, and analysed by flow cytometry as described in the legend to *Figure 3*. (*A*) CD11b expressions [median fluorescence intensity (MFI)] on circulating monocytes in control and AdAng-1 groups were compared 10 days after virus administration. (*B*) CD11b expression on Gr1^-^ and Gr1^+^ monocytes in control and AdAng-1 groups were analysed and representative histograms were depicted. **(***C***)** The correlation between MOMA-2-positive areas and CD11b expression on Gr1^+^ monocytes on day 10 after AdAng-1 administration was analysed. CD11b expression was represented by MFI. Pooled data are presented from three independent experiments (*n* = 5 per experiment). **P<0.01.

The β_2_-integrin heterodimer CD11b/CD18 (α_M_β_2_, also called Mac-1) is one of the major adhesion molecules on monocytes that mediates firm adhesion to endothelial cells *via* intercellular adhesion molecule-1 (ICAM-1).^33^^,^^34^ It is required for monocyte reverse migration and may play a role in atherosclerosis development.^35^^,^^36^ Immunostaining of ICAM1 at the sinus did not show any difference between control virus-treated and AdAng-1-treated mice (Supplementary material online, *Figure S4*). However, CC chemokine receptor 2 (CCR2) expressions on circulating monocytes showed significant increase 10 days after AdAng-1 infection (data not shown), suggesting that Ang-1 may contribute to increased monocyte recruitment. To determine whether the pro-atherogenic effects of Ang-1 are associated with changes in β_2_-integrins on monocytes, we analysed the expression of CD11b on circulating monocytes. Mice treated with AdAng-1 have a significantly lower level of monocyte CD11b expression 10 days after AdAng-1 treatment (*Figure 7A*). In addition, this Ang-1-induced down-regulation of CD11b was Gr1^+ ^monocyte specific (*Figure 7B*), and the down-regulation of CD11b persisted up to 28 days after virus administration, after expression of Ang-1 had declined (Supplementary material online, *Figure S5*). Furthermore, there was a significant negative correlation between CD11b and MOMA-2-positive lesion area in ApoE^-/-^ mice (*Figure 7C*), suggesting a possible protective function of CD11b, which is inhibited by Ang-1, thereby resulting in enhanced atherosclerosis in ApoE^-/-^ mice.

To further investigate the mechanism by which down-regulation of CD11b may reduce monocyte transmigration, we examined the interactions of monocytes with human umbilical vein endothelial cells (HUVECs). We found that blocking CD11b function with the monoclonal antibody ICRF44 reduced monocytes reverse transmigration through HUVEC monolayers by more than two-fold (IgG control; *n* = 3, transmigrated 15.70% vs. ICRF44; *n* = 3, transmigrated 32.43%, *P* = 0.068) (Supplementary material online, *Figure S6*), suggesting that down-regulation of CD11b expression on monocytes may lead to increased monocyte retention.

In addition, we explored Tie2 expression in Gr1 ^+^ CD11b^+ ^and Gr1^-^CD11b^+ ^monocytes and found that Tie2 expression was largely restricted to Gr1^+ ^monocytes compared to Gr1^-^ monocytes in both blood (Supplementary material online, *Figure S7*, 4.000 ± 0.1941 vs. 1.973 ± 0.176; *P *<* *0.01) and spleen (Supplementary material online, *Figure S7*, 10.44 ± 1.376 *vs*. 3.892 ± 0.3050; *P *<* *0.01). Immunostaining of aorta obtained from ApoE^-/-^ mice received AdAng-1 treatment revealed that Tie2-positive macrophages were present in the plaques (Supplementary material online, *Figure S8*), suggesting that Ang-1/Tie2 pathway on monocytes, is in part, responsible for Ang-1 pro-atherogenic effects.

## 4. Discussion

The activation of endothelial cells and the recruitment of monocytes are key events in the early onset of atherosclerosis; however, these initial processes may be reversible and typically do not cause clinical consequences.^1^^,^^37^ It is the subsequent prolonged retention of monocytes/macrophages in the intimal space and their uptake of oxidized LDL to form foam cells that constitutes the major cellular component in early atherosclerotic lesion development.^38^ The major finding of this study is that systemic overexpression of Ang-1 accelerates atherosclerosis development in ApoE^-/-^ mice. It has been shown that levels of Ang-1, but not of Ang-2, are significantly increased in conditioned medium from cultured atherosclerotic arteries compared to healthy arteries.^17^ It has also been shown that Tie2 expression is increased in atherosclerotic arteries compared to healthy arteries. These findings support our results and suggest that the Ang-1/Tie2 system has a significant role in the development of atherosclerosis. Although Ang-1 has been shown to have anti-inflammatory properties in endothelial cells, our present study clearly demonstrates pro-remodelling effects of Ang-1 on monocytes that translate into increased atherosclerosis in the context of elevated cholesterol levels in ApoE^-/-^ mice. The mechanisms by which Ang-1 promotes atherosclerosis appear to involve (i) Ang-1-induced pro-remodelling cytokine release; (ii) Ang-1 increases the proportion of circulating inflammatory monocytes and (iii) Ang-1-mediated reduction in CD11b expression on monocytes, resulting in increased monocyte/macrophage retention in atherosclerotic plaques.

In the present study, plasma levels of VEGF and MCP-1, both of which promote atherosclerosis in mice,^39^^,^^40^ are increased following systemic overexpression of Ang-1. Ang-1 increased VEGF and MCP-1 release via Tie2 from aortic rings suggesting that the inflammatory effects of Ang-1 are evident in large arteries, in which atherosclerotic plaques form. Taken together our data suggest that Ang-1/Tie2 pathway participates in the inflammatory process under these conditions. VEGF is a potent regulator of vascular permeability.^41^ It not only stimulates endothelial cell proliferation but also upregulates other pro-remodelling cytokines release from endothelial cells, such as MCP-1.^42^ Ang-1-induced increased levels of VEGF and MCP-1 are positively correlated with each other in both *in vivo* and *in vitro* systems, suggesting Ang-1 can create a positive pro-remodelling cytokine feedback loop in large arteries, which resulted in sustained inflammation even after systemic Ang-1 levels dropped to baseline 14 days after AdAng-1 injection (Supplementary material online, *Figure S1*).

Hypercholesterolemia induces monocytosis and monocytes accumulation in the plaques.^43^ Recently, the importance of neutrophil in early atherosclerosis development has been increasingly recognized.^44^ Doring *et al.*^43^ have demonstrated that the mechanism of neutrophil-driven atherosclerosis is mediated through neutrophil granule protein cathelicidin-induced inflammatory monocytes recruitment,^45^ suggesting that bone marrow-derived monocytes are crucial in atherosclerosis development. Hypercholesterolemia is known to induce selective expansion of Gr1^+^/Ly6C^high^ monocytes in ApoE^–/–^ mice, and these cells preferentially adhere to activated endothelium, infiltrate the arterial wall and develop into atherosclerotic macrophages.^7^ Ang-1 overexpression leads to a reduction in the prevalence of Gr1^+^/Ly6C^high^ inflammatory monocytes in bone marrow but an increase in circulation in AdAng-1-treated animals, suggesting that Ang-1 may trigger monocyte mobilization from bone marrow most likely through up-regulation of MCP-1.^32^^,^^40^^,^^46–49^ The imbalance of monocyte subsets in circulation induced by Ang-1 overexpression is associated with increased atheroma (*Figure 3D*). In addition, we show for the first time that Gr1^+^/Ly6C^high^ not Gr1^-^/Ly6C^low^ monocytes express Tie-2 in ApoE^-/-^ mice (Supplementary material online, *Figure S7*). Until recently, Tie-2 was thought to be restricted to endothelial cells. However, De Palma *et al.*^48^ identified a subset of Tie-2-positive monocytes that promote angiogenesis in experimental tumour model.^50^ Recently, studies have revealed that functions of Tie-2-expressing monocytes (TEMs) may not be restricted to angiogenesis and immunosuppression. TEMs are involved in inflammatory process.^51^^,^^52^ Our findings suggest that Ang-1/Tie2 is, in part, responsible for migration/recruitment of Gr1^+^/CCR2^+ ^monocytes in addition to MCP-1. Furthermore, the presence of Ly6C ^+^ cells within atherosclerotic plaques (*Figure 4*) provides further evidence that this group of cells is likely to account for the observed accumulation of monocytes/macrophages, which contributed to Ang-1-induced atherosclerosis development.

Adhesion molecules participating in monocyte–endothelial cell interactions are known to play a critical role in atherogenesis.^35^ In this study, we observed that overexpression of Ang-1 increased oil red-positive lesion size and it is associated with down-regulation of CD11b expression on circulating Gr1^+ ^monocytes. It has become increasingly clear that the dynamic trafficking of monocyte-derived cells within atherosclerotic lesions is closely linked to disease progression.^53^ Prolonged retention of monocytes/macrophages in the intimal space^1^^,^^2^ or reduced rate of mononuclear cell emigration (i.e. reverse migration) from lesions^53^ is crucial for atherosclerosis development. Reverse migration is a physiological feature of human mononuclear phagocytes^54^ and that this process is dependent on both ICAM-1 and CD18 (integrin β_2_).^54^ Since CD11b forms functional heterodimer complex with CD18, down-regulation of CD11b expression is likely to affect monocyte reverse migration and as a consequence would favour monocyte retention and progression of atherosclerosis. Our assumption is supported by *in vitro* study showing that blockade of CD11b function leads to reduced monocyte reverse transmigration through endothelial cells and inverse correlation between CD11b expression and plaque monocyte/macrophage accumulation (*Figure 7C*). Merched *et al.*^53^ showed that β2 integrin deficiency accelerates early atherosclerosis in LDLR^−/− ^mice, suggesting that CD11b may play a dynamic role in the development of atherosclerosis. Interestingly, CD11b/CD18 activation has been proven to be able to inhibit macrophage lipid uptake, and CD11b-deficient peritoneal macrophages have up-regulated level of CD36 expression, and lipid accumulation compare to wild-type controls,^54^ suggesting that Ang-1-induced down-regulation of CD11b may contribute to lipid accumulation in the lesions.

In conclusion, Ang-1 overexpression induces MCP-1 and VEGF in circulation and subsequently caused inflammatory monocyte mobilization, and down-regulated CD11b expression on these cells, leading to monocytes/macrophages accumulation and atherosclerotic plaque formation. Our findings provide a novel mechanism by which Ang-1 may contribute to atherosclerosis development. 

## Supplementary material

Supplementary material is available at *Cardiovascular Research* online.
